# Supplementary material for: Practice variation in induction of labor: A critical document analysis on the contribution of regional protocols
Source: PLoS One. 2024 Oct 1;19(10):e0311032. doi: 10.1371/journal.pone.0311032 (PMC11444378; doi:10.1371/journal.pone.0311032)
Supplement: S1 Appendix — (DOCX) [file pone.0311032.s001.docx]

| **Subject** | **Questions** |
| --- | --- |
| Content regional protocol | 1. What indicates that the regional protocol contributes to more or fewer inductions of labor (IOL) compared to the recommendations in national guidelines?    1. What other interventions are described that may influence the number of IOL? Are any preventive interventions described that may affect more or less midwife led care?    2. What (scientific) basis is there for these recommendations? (national guideline, additional literature research, other?) |
| Women’s involvement | 1. What is described about women’s involvement in clinical decision-making? |
| National guideline vs regional protocol | 1. What is the quality of the national guideline according to the AGREE assessment? Is there a relationship between the quality of the national guideline (AGREE) and the existence of variation between regional protocols? |
| Development procedure | Questions at Maternity Care Network (MCN) level:   1. What is the process to develop regional protocols/work arrangements? Issues to consider:    1. Does the regional protocol describe only midwife-led care, obstetrician-led care, or complete integrated care?    2. How were the different disciplines involved in developing and approving the regional protocol?   Points of interest: role of the primary care midwives, clinical midwives, obstetricians, junior doctors. Which disciplines are represented?   - 1. How is the implementation of the regional protocol supported? What is the follow-up process like? (review, accessibility) |
| Overall impression | What is your overall impression of this regional protocol/work arrangement at maternity care of this MCN?  Points of interest:   - Manner and responsible caregiver for counselling - How does this regional protocol affects cooperation? - Is there difference between the rationale (background/substantiation) in the protocol and the work arrangements of the protocol? - Does the regional protocol fit with an MCN with a high or low group for IOL for low-risk pregnancies? |

**S1 Appendix. Analytical framework for analyzing regional protocols.**
